# Effects of Cadmium on Physiochemistry and Bioactive Substances of Muskmelon (*Cucumis melo* L.)

**DOI:** 10.3390/molecules27092913

**Published:** 2022-05-03

**Authors:** Yunqiang Wang, Zhen Lei, Rongbin Ye, Wei Zhou, Ying Zhou, Zhengkang Zou, Junli Li, Licong Yi, Zhaoyi Dai

**Affiliations:** 1 Institute of Economic Crops, Hubei Academy of Agricultural Science, Wuhan 430064, China; wangyunqiang0909@sina.com (Y.W.); zhouwei426@hbaas.com (W.Z.); ylc881128@163.com (L.Y.); 2 Vegetable Germplasm Innovation and Genetic Improvement Key Laboratory of Hubei Province, Hubei Academy of Agricultural Science, Wuhan 430064, China; 3 School of Chemistry, Chemical Engineering and Life Sciences, Wuhan University of Technology, Wuhan 430070, China; lz1791250229@163.com (Z.L.); wasdjkl24682022@163.com (R.Y.); zhouying981012@163.com (Y.Z.); zzk1322165547@163.com (Z.Z.)

**Keywords:** cadmium pollution, muskmelon pedicel, bioactive substances, health risks

## Abstract

Muskmelon pedicel is the fruit stalk of muskmelon and one of the traditional Chinese medicines, which can be used to treat jaundice, diabetes and neuropathy. However, in recent years, agricultural soil heavy metal cadmium (Cd) pollution has become serious, coupled with the imperfect sales management of herbal medicine, increasing the potential health risk of contaminated herbal medicine in the human body. In this paper, the comprehensive quality of contaminated muskmelon was tested. The results showed that Cd stress significantly inhibited the growth of muskmelon plants, reduced the anthocyanin and chlorophyll contents, and increased the fruit size and sweetness of muskmelon. In addition, heavy metal Cd can also cause oxidative stress in plants, resulting in a series of changes in antioxidant enzyme activities. In the experimental group, the content of polyphenols and saponins increased by 27.02% and 23.92%, respectively, after high-concentration Cd treatment, which may be a mechanism of plant resistance to stress. This paper reveals that the content of bioactive substances in Chinese herbal medicine is high, but the harm in heavy metals cannot be underestimated, which should be paid attention to by relevant departments.

## 1. Introduction

Many herbs and their preparations have been widely used in China, South Korea, Japan, and many other eastern countries for thousands of years. Natural products are accepted by people, and people’s interest in natural products has increased significantly, in developing countries and developed countries, in recent years. In 2014, of cancer drugs in the market, 49% were extracted from natural products [1,2,3]. Muskmelons (*Cucumis melo* L.) are members of the gourd family and are widely grown worldwide in temperate, subtropical, and tropical regions. The leading muskmelon producers are China, the United States, Spain, Turkey, and Iran. Muskmelons are also considered an important source of natural plant products, and the physical, biochemical, and phenotypic characteristics of muskmelons vary greatly depending on climate regions and local preferences [4,5]. The muskmelon pedicel (*Pedicellus melo*) is the dried fruit stalk of muskmelon and one of the traditional Chinese medicines, which can be used to treat jaundice, diabetes, neuropathy, to improve immunity, anti-inflammatory properties, and so on [6]. Due to the high content of cucurbits, they are used in traditional Chinese medicine, such as liver medicine made from the pedicel. However, polyphenols, flavonoids, and sugars account for a large proportion of the research on the components of the bioactive substance in muskmelon. The physiological, biochemical, and bioactive substance components of muskmelon were studied in this paper [7,8,9].

According to the National Soil Pollution Survey Bulletin released by the Ministry of Environmental Protection and the Ministry of Land and Resources in 2014, some soils in Chinese industrial and agricultural areas are polluted by heavy metals, and cultivated land resources are decreasing day by day. The exceeding standard rate of heavy metal pollution in an industrial area and the surrounding soil was 36.3%, and the exceeding standard rate of cultivated land was 19.4% [10,11]. Among metals, Cd is considered highly toxic to organisms and humans. The development of agriculture and industry eventually resulted in an increase in Cd concentrations of agricultural soils. At the same time, Cd is accumulated in plants through agricultural soil, entering the food chain and accumulating in various organs after entering the human body, causing serious health hazards [12,13]. Heavy metal Cd not only accumulates in plants but also has significant effects on plant growth and physiology. Studies have shown that heavy metal Cd in the soil is taken up by plant roots and transported up to other parts of the plant [14]. In other studies, plants defend against Cd stress by increasing superoxide dismutase (SOD), catalase (CAT), and peroxidase (POD) [15].

Plants are also widely used in the treatment of heavy metals. For example, in Giannakoula’s study, citrus had some detoxification and resistance [16,17]. However, crops grown in heavy metal soils also have potential health risks, especially if they are not known to have been grown in heavy metal soils [18]. Especially in western countries, herbal medicines are often sold as dietary supplements and are available on the market without the necessary evidence of efficacy and safety. The global market is generally not standardized in the sales of herbs. Consumers can buy herbs through various channels, not only in pharmacies but also in online stores [19,20,21]. In order to deceive consumers, many merchants use wrong or false origin information to impersonate the origin of herbal medicines. For example, the factory is located in an industrial estate, where heavy metal pollution is serious, which greatly increases the adverse reactions of herbs. Sellers make huge profits from counterfeit and inferior products, which is worthy of the attention of the relevant departments and us. Therefore, the quality inspection of medicinal materials is very important to the quality management of medicinal materials [22,23,24].

There is no unified standard for the quality evaluation of natural products in the world, and there are endless quality testing methods for herbal medicines, such as chemical methods, physical methods, biological methods, and the relatively new fingerprint method [25,26,27]. For example, the DNA barcoding technique developed by Mishra and colleagues for quality control of medicinal materials [28]. However, few people have evaluated the quality of herbal medicines comprehensively from the physiological and biochemical examination of the plants from the origin of herbal medicines, as well as the detection of basic heavy metals. In our study, it was found that under the stress of heavy metal Cd, the bioactive substances of muskmelon and fruit sugar increased, which may be used by undesirable vendors, thus polluting the herbal medicine in the human food chain. Therefore, comprehensive quality testing of polluted melons is of great significance for food safety protection for consumers. The basic physiology and biochemistry of muskmelon were tested, and the self-protection mechanism of muskmelon under Cd stress was explored by the activity of antioxidant enzymes. The potential health risk of polluted muskmelon parts was evaluated by the Cd concentration of each part. In this paper, the extraction method and content of bioactive substances from pedicel were studied, the cytotoxicity of extracts from pedicel was tested through pharmacological experiments, and the mechanism of Cd on the increase of bioactive substances from pedicel was discussed.

## 2. Results and Discussion

### 2.1. Physiological Indexes and Fruit Quality

**Figure 1:**
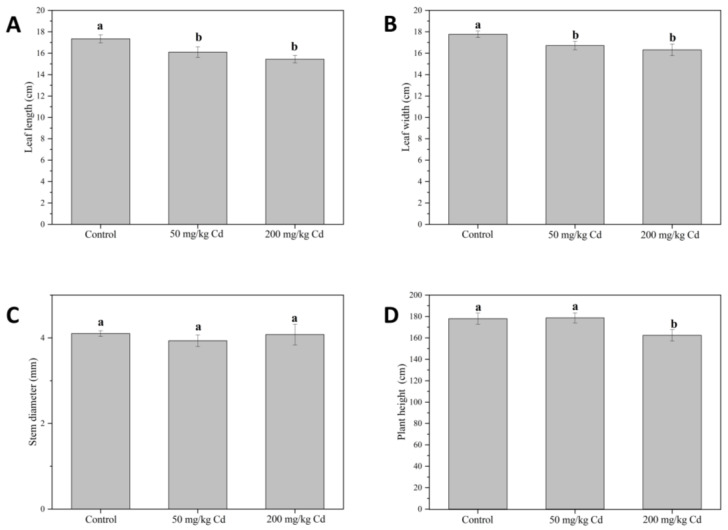
Leaf length (**A**), leaf width (**B**), stem diameter (**C**), and plant height (**D**) of muskmelon plants in different treatment groups. The data were three repeated means ± standard deviation. Different lowercase letters indicate significant differences at *p* < 0.05.

As can be observed in Figure 1, Cd treatments significantly reduced leaf length (Figure 1A) and leaf width (Figure 1B) but had no significant effect on stem diameter (Figure 1C). A Cd stress of 50 mg/kg had no significant effect on muskmelon plant height (Figure 1D), while 200 mg/kg Cd treatment significantly reduced muskmelon plant height by 8.75%. In general, Cd stress had a significant inhibitory effect on the growth of muskmelon plants, and as the Cd concentration increases, the inhibitory effects become more noticeable. Growth inhibition is part of the distinct symptoms of metal toxicity. It is reported that Cd has a serious impact on plant growth [13,29]. In addition, there was no significant change in stem diameter under Cd treatment, which may be due to the different transport and accumulation of organs under heavy metal stress or the detoxification mechanism of plants themselves. Cd accumulation often leads to visible plant symptoms, such as growth inhibition or leaf yellowing [30,31]. In the study of Monteiro, Cd has different cytotoxicity in roots and leaves, which is due to different antioxidant pathways to different parts, and the accumulation of Cd in roots is greater than that in leaves [32].

Even after being treated with Cd, the muskmelon plants still bore fruit. From the perspective of fruit quality (Table 1), compared with the control, the two concentrations of Cd significantly increased the fruit weight, longitudinal diameter, transverse diameter, and flesh thickness of muskmelon, but there was no significant difference in fruit quality between the two Cd treatments. Compared with non-Cd treatment, under 50 mg/kg Cd stress, the weight, vertical diameter, transverse stems, and flesh thickness of muskmelon fruit increased by 19.118%, 10.255%, 7.201%, and 6.194%, respectively. At the same time, under 200 mg/kg Cd stress, the weight, vertical diameter, transverse stems, and flesh thickness of muskmelon fruit increased by 14.216%, 8.772%, 5.435%, and 10.816%, respectively. Different Cd concentrations increased the soluble sugar content of muskmelon. In general, 50 and 200 mg/kg Cd treatments increased the size of the muskmelon fruit and increased the sugar content of the muskmelon fruit. Whether the fruits treated with heavy metals can enter the human body through the food chain concerns consumers [33]. When consumers buy melons, they will not only consider the appearance of the melon but also consider the sweetness and maturity of the fruit. In addition, Cd-treated muskmelons with higher sugar content are more likely to be chosen by consumers [34,35].

### 2.2. Detection of Physiological Indexes

**Figure 2:**
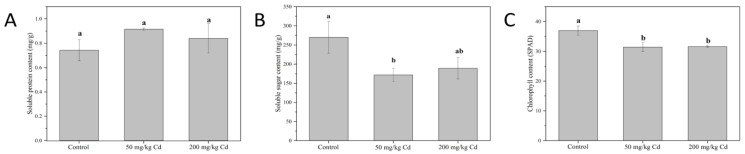
The content of soluble protein (**A**), soluble sugar (**B**), and chlorophyll (**C**) of muskmelon plants in different Cd concentration treatment groups. The data show the means ± SD of three replicates. Different lowercase letters indicate significant differences at *p* < 0.05.

The soluble protein, soluble sugar, and chlorophyll of melons were detected. Soluble protein is the main osmotic adjustment substance in plants, which contains a variety of enzymes related to plant metabolism, and is often used as an index for screening resistance [36,37]. As shown in Figure 2A, Cd treatment did not have a significant effect on the soluble protein content of muskmelon plants. Soluble sugar can be used as osmotic adjustment substances in plants, as well as nutrients and structural substances, so it is also one of the indicators for screening resistance [38]. As shown in Figure 2B, 50 and 200 mg/kg Cd treatments reduced the soluble sugar content of muskmelon by 35.94% and 29.73%, respectively. This may be related to the significant decrease in chlorophyll content of muskmelon plants under Cd stress, which affects the photosynthesis of plants [39]. Chlorophyll is inseparable from the photosynthesis of plants, and it bears the important task of capturing light energy in photosynthesis [40]. As shown in Figure 2C, the 50 and 200 mg/kg Cd treatments significantly reduced the chlorophyll content of muskmelon plant leaves by 14.94% and 14.57%, respectively. The reduction of chlorophyll content will further affect the photosynthesis of plants, which will adversely affect the growth of muskmelon, which is consistent with the results of the growth indicators recorded previously.

### 2.3. Antioxidant System

Plants will produce oxidative stress when exposed to external pressure and produce a large number of reactive oxygen species (ROS) in the body [41,42]. ROS can interact with proteins, lipids, and nucleic acids in cells, thereby changing their structure and function, which may eventually lead to lipid peroxidation, enzyme inactivation, and DNA fragmentation and mutation [43,44,45]. Under normal circumstances, plants can regulate the active oxygen in the body in a dynamic balance through the non-enzymatic systems (abscisic acid, anthocyanins, etc.) and enzyme systems (antioxidant enzymes SOD, POD, CAT, APX, etc.) in vivo [46,47,48].

**Figure 3:**
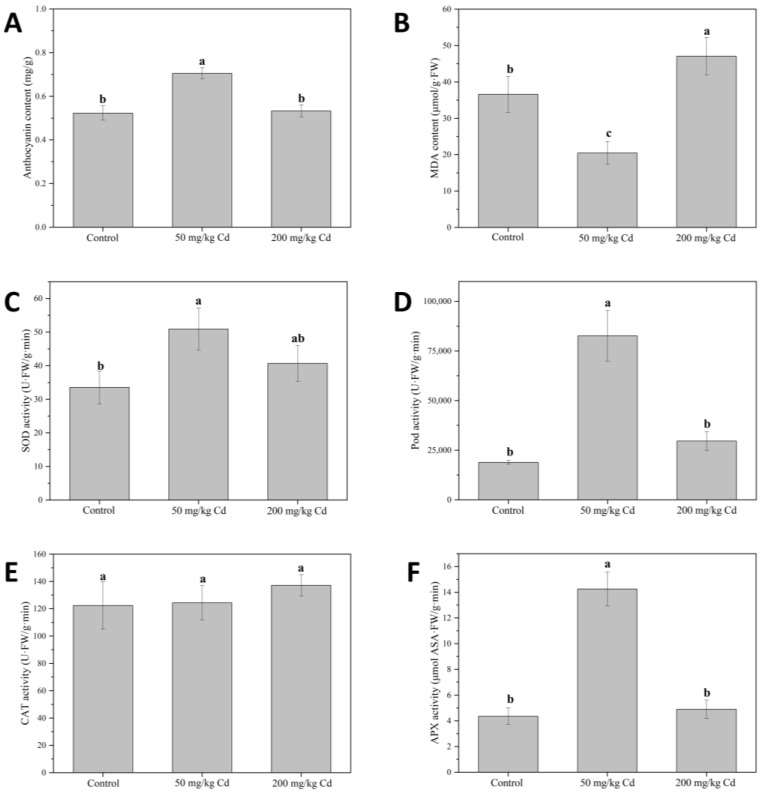
Muskmelon anthocyanin (**A**) and MDA (**B**) content, and SOD (**C**), POD (**D**), CAT (**E**), and APX (**F**) activity in different concentrations of Cd treatment groups. Different lowercase letters indicate significant differences at *p* < 0.05.

Anthocyanins are key pigments in plants, closely related to plant signal transmission [49]. Studies have shown that biotic and abiotic stresses, such as pathogen infection and heavy metal pollution, can cause the accumulation of anthocyanins in plants [50,51]. As shown in Figure 3A, when treated with 50 mg/kg Cd, the anthocyanin content of muskmelon leaves significantly increased compared with the control, indicating that 50 mg/kg of Cd produces oxidative stress in the muskmelon plants [52].

SOD can convert superoxide anions into H_2_O_2_, and CAT, POD, and APX can decompose H_2_O_2_ into water and oxygen, thereby alleviating the toxicity of reactive oxygen species to cells [53,54]. As shown in Figure 3C, 50 mg/kg Cd treatment group significantly increased the SOD activity of muskmelon plant leaves by 51.76% compared to the control. When the Cd concentration reached 200 mg/kg, the SOD activity showed a downward trend but was still higher than the control group. In the results, POD (Figure 3D) and APX (Figure 3F) activities were similar to those of SOD; both significantly increased under 50 mg/kg Cd treatment but decreased under 200 mg/kg Cd treatment. There was no significant difference in the activity of CAT (Figure 3E) under the two concentrations of Cd treatment compared with the control. This implies that under the treatment of a lower concentration of Cd, the active oxygen content in the plant increases, and the plant enhances the antioxidant response in the body by inducing the synthesis of anthocyanins and the activity of SOD, POD, and APX. When the concentration of Cd increases to 200 mg/kg, the excessive active oxygen produced in the body may not be removed, causing damage to the cells and irreversible damage to the antioxidant system of the plant, SOD, POD, and APX activity is reduced. When the excessive reactive oxygen species in plants cannot be removed in time, it may lead to cell lipid peroxidation, thereby causing membrane leakage [55,56]. MDA can be used as an indicator of planting cell lipid peroxidation [57]. As shown in Figure 3B, the MDA content of the 200 mg/kg Cd treatment group was significantly higher than that of the control group, indicating that due to the high concentration of Cd stress, lipid peroxidation may occur in the plant, while for the plant cells under the 50 mg/kg Cd treatment, the MDA content did not increase, indicating that the Cd at this concentration is not enough to cause lipid peroxidation in muskmelon plant cells [58]. CAT activity did not change significantly under 50 and 200 mg/kg Cd treatments, which may be due to the fact that CAT was not as sensitive to Cd as other antioxidant enzymes and had little effect on scavenging reactive oxygen species. The research on herbs mainly focuses on the effective components of some herbs. In addition, due to different harvest seasons, habitats, drying processes, and other factors, the chemical components of medicinal materials were also different. In the production chain of herbal medicine, the growth conditions and harvest time of herbal medicine will affect the quality of the final herbal medicine. In order to comprehensively monitor the quality of herbal medicine, physiological and biochemical indexes before harvesting are necessary for the quality control of herbal medicine [25,59].

### 2.4. Cd Content

**Figure 4:**
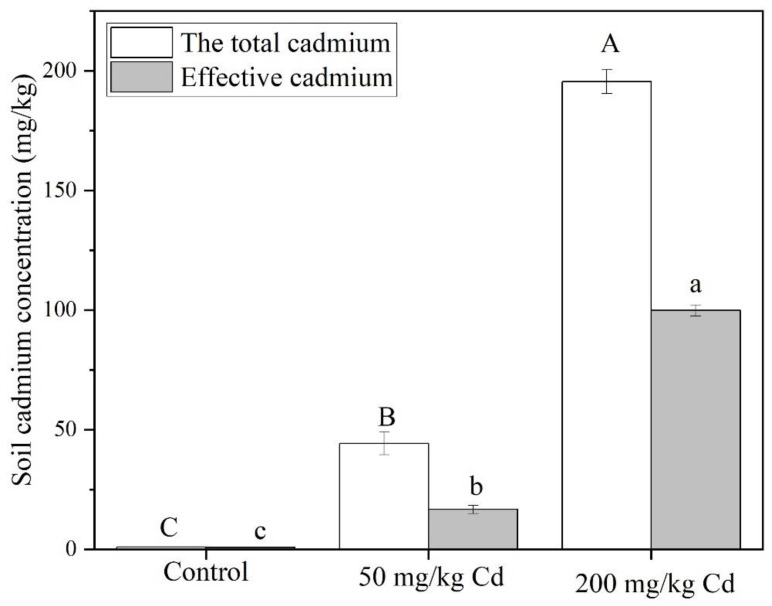
Total Cd and available Cd content of soil in different Cd concentration treatment groups. The data show the means ± SD of three replicates. Different uppercase and lowercase letters show significant differences, *p* < 0.05.

In an aqueous solution, Cd usually appears in the form of a divalent Cd^2+^ cation, while heavy metals exist in the soil in five forms: exchange state, carbonate state, iron and manganese oxidation state, organic binding state, and residual state. In this paper, the Cd treatment group is achieved by adding Cd chloride into the soil and mixing uniformly [60,61]. As shown in Figure 4, both the total Cd and the effective Cd concentration in the soil of the 200 mg/kg treatment group were significantly greater than the 50 mg/kg treatment group. The total Cd concentration of all treatment groups was superior to the effective Cd concentration. In the 50 mg/kg treatment group, the total Cd concentration was 2.65 times bigger than the effective Cd concentration, while it was 1.96 times bigger than that in the 200 mg/kg treatment group.

**Figure 5:**
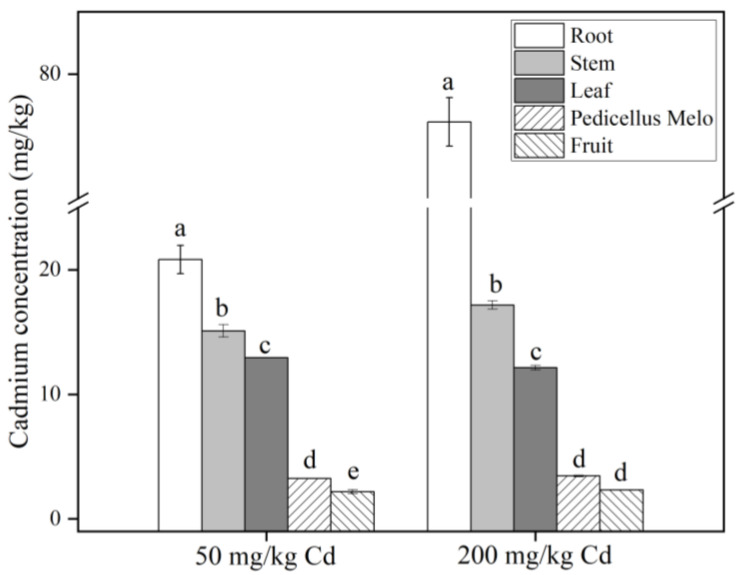
Cd content in roots, stems, leaves, muskmelon pedicel, and fruits of muskmelon plants under different Cd treatments. The data show the means ± SD of three replicates. The concentration of Cd was set to zero for the no Cd treatment, and different lowercase letters indicate significant differences at *p* < 0.05.

As shown in Figure 5, regardless of 50 or 200 mg/kg Cd treatments, the Cd content in each part of the plant is roots > stems > leaves > muskmelon pedicels > fruits. The Cd content in roots, stems, and pedicels was significantly higher in the 200 mg/kg treatment group than in the 50 mg/kg treatment group. In the food and grain sector, China’s cadmium concentration limit standard is 0.2 mg/kg, while the World Health Organization’s limit for cadmium is 0.4 mg/kg [62]. In our experiment, the Cd concentration of 50 mg/kg Cd in melon fruit and pedicel was 2.191 and 3.235 mg/kg, respectively, both of which were far beyond the prescribed standards. The harm of heavy metal Cd pollution should not be underestimated. Therefore, it is necessary to evaluate the health risks of Cd-treated muskmelons.

The intake rate of muskmelon is 12.01 g per person per day according to the intake rate of watermelon, and the weight of a person is counted as 60 kg [63]. Other necessary parameters are calculated according to international regulations, as shown in Table 2. Under the same Cd concentration, the threshold hazard quotient (THQ) and the threshold carcinogenic risk (TCR) of different parts were significantly different. If the THQ is less than 1, there is no adverse health effect. On the contrary, if THQ is greater than 1, it indicates a potential non-carcinogenic effect. Under two cadmium concentrations, the risk coefficients of all parts except roots were greater than 1. In addition, THQ greater than 1 indicates that it may have a potential non-carcinogenic effect. A cancer risk assessment was then performed, and TCR was a recognized parameter used to determine an individual’s incremental risk of developing cancer over his or her lifetime. However, when TCR is greater than 0.0001, it indicates the potential carcinogenic risk. In our study, even the fruit treated with 50 mg/kg is far more than 0.0001, indicating the potential carcinogenic risk of different parts. It is worth noting that our experiment was artificially set heavy metal content, while under normal environments, Cd concentration is very low in the condition of no Cd treatment, far below the limit of carcinogenicity risk [64,65].

### 2.5. The Content of Bioactive Substances

#### 2.5.1. Optimization of Extraction Process

According to the results of the single-factor experiment (Tables S2–S5), an orthogonal experiment L9 (_3_^4^) with four factors and three levels was designed. The experimental results are shown in Table S6, where A, B, C, and D represent the material-to-liquid ratio, extraction time, ethanol concentration, and extraction temperature, respectively.

As shown in Table S7, according to the R-value, the order of factors affecting the content of polyphenols in the extract was as follows: ethanol concentration > extraction temperature > solid-liquid ratio > extraction time. Due to the different combinations of optimal extraction conditions for different extracts, comprehensive consideration is needed. A comprehensive weighted scoring method is used to determine the optimal extraction conditions based on polyphenols, flavonoids, and saponins accounting for 30 and polysaccharides accounting for 10. Weighted factor coefficient bid= score/range. According to Table S6, the determined range (maximum concentration–minimum concentration in the orthogonal table) of polyphenols, flavonoids, saponins, and polysaccharides were 0.002, 0.090, 0.048, and 58.515, respectively. After calculation, the weighting factor coefficients of polyphenols, flavonoids, saponins, and polysaccharides are bi1 = 17602.118, bi2 = 333.414, bi3 = 627.022, bi4 = 0.171. Comprehensive score yi = yi1 × bi1 + yi2 × bi2 + yi3 × bi3 + yi4 × bi4 [66]. The comprehensive score of each experiment is shown in Tables S6 and S7. Under certain conditions (ethanol concentration (A), temperature (B), liquid-solid ratio (C), time (D)), the average yield (k) of a certain level is the largest; that is, the optimal extraction power. The range (R) represents the difference between the maximum yield and the minimum yield. The larger R-value represents the main influencing factor of the extraction process [67,68]. According to Table S6, the combination with the highest overall score is number 4, namely A2B1C2D3. That is, the optimal extraction scheme is as follows: extraction temperature is 80 °C, the solid-liquid ratio is 1:40, ethanol concentration is 80%, and the extraction time is 4 h.

#### 2.5.2. Effective Components Content of Muskmelon Pedicel

**Figure 6:**
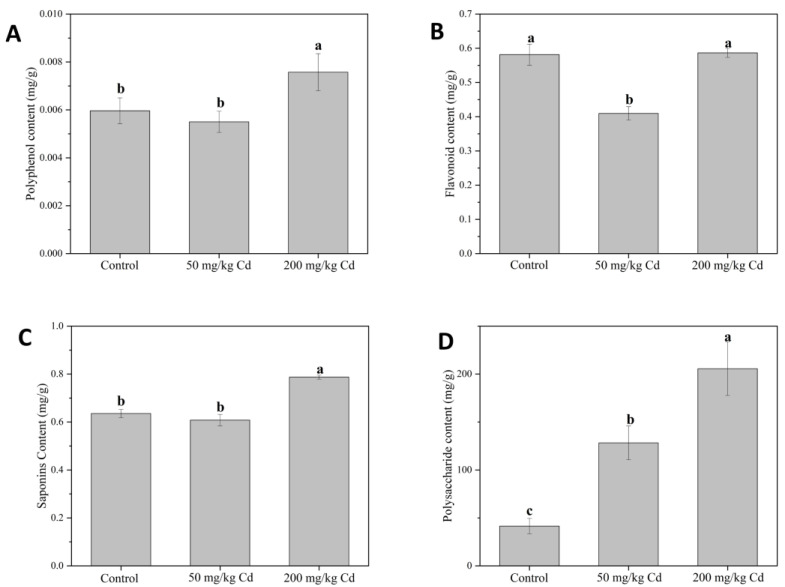
The contents of polyphenols (**A**), flavonoids (**B**), saponins (**C**), and polysaccharides (**D**) in muskmelon plants under different concentrations of Cd. The data show the means ± SD of three replicates. Different lowercase letters indicate significant differences at *p* < 0.05.

In our experiment, the contents of polyphenols, flavonoids, and saponins were inhibited at 50 mg/kg Cd but increased under a high concentration of Cd. Regarding the polyphenol content (Figure 6A), 50 mg/kg Cd treatment had no significant effect on the polyphenol content in the pedicel, while the 200 mg/kg Cd treatment significantly increased the polyphenol content in the pedicel. Compared with the control group, it significantly increased by 27.02%, indicating that Cd treatment can increase the polyphenol content in the pedicel. Polyphenols accumulate in response to different abiotic stresses that plants need to withstand, helping plants adapt to adverse conditions. The increased antioxidant capacity of plants is related to a variety of functions of polyphenols in plants, mainly including their ROS scavenging ability the protective ability of certain polyphenols. Plants grown under abiotic stress have the ability to biosynthesize more phenolic compounds than plants grown under normal conditions [69].

For the content of flavonoids (Figure 6B), 50 mg/kg Cd treatment significantly reduced the flavonoid content in the pedicel, which was 29.54% lower than that in the control group. It is shown that a low concentration of Cd inhibits the synthesis of flavonoids. Zhang found that *Robinia pseudoacacia* can improve the protection and defense system of seedlings in Cd-contaminated soil by promoting the synthesis of total flavonoids. In addition, other studies have shown that polyphenols and brass flavonoids have a rise under abiotic stress and are major sources of antioxidants [70,71]. The content of saponin (Figure 6C), similar to the content of polyphenols, was considerably increased by 23.92% compared with the control group. It shows that a minimal concentration of Cd has no significant effect on the content of pedicel, while high concentration of Cd will increase the content of saponins in muskmelon pedicel. In Liao’s research, water stress can affect plant growth and secondary metabolites, improve the expression of key genes in the pathway of notoginseng saponins synthesis, and improve the content of notoginseng saponins [72].

Compared with the control group, the content of polysaccharides in 50 and 200 mg/kg Cd treatments significantly increased by 2 and 4 times, respectively (Figure 6D). It can be seen that Cd treatments have the most significant effect on the polysaccharide content of pedicel; even at a low concentration, it can also significantly promote the increase of polysaccharide content. Soluble sugar and polysaccharides are different biological concepts. Soluble sugar generally includes glucose, fructose, and sucrose, which are the main sweeteners in fruits [73]. Polysaccharides are a kind of biomacromolecule soluble in water, and their structure is complex and diverse [74]. The two extraction methods are different and cannot be compared. However, in this study, the soluble sugars in leaves and pulp sugars showed different concentrations with the increase of Cd concentration. The soluble sugars in leaves decreased with the increase of Cd concentration, while the soluble sugars in fruits were the opposite. Because the leaf is the main sugar provider under fruit photosynthesis, when the soluble sugar in the leaf decreases, it is a plant that distributes nutrients to other important organs under Cd stress. These sugars may also be used for fruit growth and improve fruit quality because, in our experiment, the soluble sugar in the fruit, the polysaccharide in the pedicel, and the fruit size increased [75,76,77,78].

### 2.6. Anti-Tumor Activity of Muskmelon Pedicle Extract

**Figure 7:**
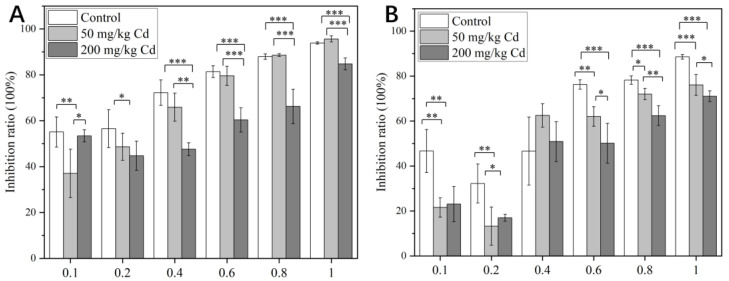
The inhibitory effects of different drug concentrations on HeLa cells (**A**) and L929 cells (**B**). The data show the mean ± SD of three replicates. * means *p* < 0.05, significant; ** means *p* < 0.01, very significant; *** means *p* < 0.001, extremely significant. The drug concentration represented by the pedicel was mg/mL, and the ordinate represented the cell inhibition rate. The greater the value, the greater the cytotoxicity.

According to the research by Gao, the therapeutic window for intragastric administration of pedicel extract in mice was 4–400 mg/kg, and there was no mortality after 14 consecutive days of administration [79]. According to the previous therapeutic window, we designed a drug gradient from 0.1 to 1 mg/mL after pre-experiment adjustment. It can be seen from Figure 7 that under the treatment of 50 and 200 mg/kg Cd, the cytotoxicity of the crude extract of pedicels to normal cells and cancer cells was generally lower than that of the control group. In the absence of Cd treatments, the crude extract of pedicel showed selectivity of HeLa cells and L929 cells, and the inhibition rate of crude extract on HeLa cells was higher than that of normal cells at a low concentration. Under the treatment of 50 mg/kg Cd, the inhibition rate of crude extract on HeLa was higher, which might be caused by the increase in some active components. Under the treatment of crude extract, the cells were concentration-dependent; that is, the inhibition rate increased with the increase of drug concentration. There were considerable differences between the treatment without Cd and the treatment with Cd.

Since ancient times, people have tended to screen melon fruit for more mature, more sweet fruit [75,76]. At the same time, in the choice of herbs, the content of effective active ingredients or bioactive components, low toxicity is the main factor [77,78,80]. However, when illegal businessmen conceal or lie about product information, factories in the original contaminated areas will bring immeasurable health hazards to consumers. Therefore, the management departments of medicinal materials and food need to improve the quality detection of functional plants.

## 3. Materials and Methods

### 3.1. Experimental Design and Source of Materials

Muskmelon (*Cucumis melo* L.) seeds were obtained from the Hubei Academy of Agricultural Sciences, Wuhan, China. CdCl_2_·2.5H_2_O (purity 99.0%) was purchased from Sinopharm Chemical Reagent Co. Ltd. (Shanghai, China). The selection of matrix soil was from Zhenjiang Peilei Matrix Technology Development Co., Ltd. (Zhenjiang, China). The temperature of the seedling bed was maintained at about 28 °C before excavation. When the seedling leaves began to grow, the temperature of the seedling bed was maintained at about 25 °C in the daytime and the lowest temperature at night was not less than 18 °C. The seedling age was 25–30 days, seedlings with 3 leaves and 1 heart were suitable for the maximum seedling age [81]. Then, the seedlings of the same size were transplanted into the flowerpot of 2.5 kg of substrate soil. In a greenhouse of the Hubei Academy of Agricultural Sciences, the light is natural light. Under natural conditions, water was provided for the plants every three days. The physical and chemical properties of the soil are shown in Table S1. Samples were taken at melon growing and fruiting stages. Sampling was performed during muskmelon growth and fruiting. The leaves at the same node position of muskmelon were collected during the growth period to analyze physiological and biochemical characteristics. Meanwhile, the plant height, stem diameter, leaf length, and leaf width were detected during the growth period. During the fruiting period, root, stem, leaf, fruit, pedicel, and soil were collected for Cd content detection. After the pedicel was dried and crushed, it was extracted to complete the cytotoxicity experiment. The actual planting image is shown in Figure S1. The contents of nitrogen, phosphorus, and potassium in the soil were determined by soil nutrient TPY-8A (Zhejiang Top Cloud-Agri Technology Co., Ltd., Hangzhou, China). The matrix soil had different concentrations (50 and 200 mg/kg) of Cd and control with no Cd previously added. Under soil culture conditions, the stress phenomenon of muskmelon could be caused without large-scale death at this concentration [82].

### 3.2. Record of Growth Indicators

The leaf length and leaf width of muskmelon plants were measured with a ruler, the plant height was measured with a tape measure, and the stem thickness was measured with a vernier caliper. The fresh weight of the muskmelon fruit was measured using an electronic scale. The horizontal diameter, vertical diameter, and flesh thickness of the muskmelon fruit were measured with a ruler. The sugar content of muskmelon fruit was determined by PAL-1 sugar detector (Japan ATAGO Co., Ltd., Tokyo, Japan).

### 3.3. Physiological Index Measurement

The 600 mg melon leaves were cut into small pieces by scissors and then added to a suitable amount of phosphate buffer (pH 7.8) to grind quickly on the ice in the mortar until there were no leaves and fibers in the solution. The grinding solution was poured into a 10 mL centrifuge tube and centrifuged at 4000 rpm for 10 min. The supernatant was taken and diluted to 10 mL with phosphate buffer to obtain the extract. The soluble protein content in melon leaves was determined by Coomassie brilliant blue G250 [83]. The absorbance of soluble protein was measured by a UV-visible spectrophotometer (Mei An (China) Instrument Co., Ltd., Shanghai, China) at 590 nm under the action of Coomassie brilliant blue. The soluble sugar content in the extract was measured using anthrone reagent [84]. The absorbance of soluble sugar was detected at 625 nm. The chlorophyll content was measured using the SPAD-502 Plus chlorophyll meter (Konica Minanda Optical Instruments (Shanghai) Co., Ltd, Shanghai, China) in three different positions of the corresponding leaves of each muskmelon plant.

Anthocyanin is colored in an acidic solution, and its color depth is proportional to the concentration of anthocyanin. The absorption wavelength of anthocyanin acid solution is 530 nm, but some extracts often contain chlorophyll, which interferes with the determination and removes the absorption wavelength of chlorophyll at 657 nm. The absorbance was measured at 530 and 657 nm, respectively. Anthocyanin concentration (mg/g) = [(A530 − 0.1 × A657 × Cyanidin-3-glucoside molecular weight)/(Cyanidin-3-glucoside molar extinction coefficient × colorimetric dish path (1 cm)) ] × extract volume (mL)/sample weight (g) [85]. The content of malondialdehyde (MDA) was determined by the reaction of malondialdehyde and thiobarbituric acid (TBA). MDA content was determined by UV spectrophotometer at 450, 532, and 600 nm. MDA content (μmol/g · FW) = [6.45 × (A532 − A600) − 0.56 × A450] × total volume of reaction system (mL) × total volume of extract (mL)/determination of extract dosage (mL) × sample fresh weight (g) [86]. The activity of SOD was measured based on its reaction with riboflavin, and nitrogen blue tetrazole SOD activity was measured at 560 nm. SOD activity (U FW/g·min) = (absorbance of phototube − absorbance of sample tube) × total enzyme solution (mL)/phototube absorbance/0.5/leaf fresh weight (g)/enzyme solution (μL) [87]. The activity of POD was determined based on its ability to oxidize guaiacol to the brown substance in the presence of hydrogen peroxide (H_2_O_2_). The absorbance of the brown substance was measured at 470 nm within a certain time, and the POD activity was calculated according to the slope of absorbance and time. POD activity (U·FW/g·min) = (absorbance of reference tube − absorbance of sample tube) × total volume of sample solution (mL)/0.01 × sample dosage (mL) × reaction time (min) × fresh weight of sample (g) [88]. CAT activity was measured based on its ability to catalyze the decomposition of H_2_O_2_ into water and molecular oxygen. The absorbance of the reactant was measured at 240 nm within a certain time, and the CAT activity was calculated according to the slope of absorbance and time. CAT activity (U·FW/g·min) = (absorbance of reference tube − absorbance of sample tube) × total volume of sample solution (mL)/0.1 × sample dosage (mL) × reaction time (min) × fresh weight of sample (g)) [89]. Ascorbic acid peroxidase (APX) activity was measured by its ability to catalyze the reaction between ascorbic acid (ASA) and H_2_O_2_, respectively. The absorbance of residual ASA was measured at 290 nm within a certain time, and ASA activity was calculated according to the slope of absorbance and time. APX activity (μmol AsAFW/g·min) = (absorbance of reference tube − absorbance of sample tube) × total volume of sample solution (mL) × total volume of reaction (mL)/2.8 × sample dosage (mL) × reaction time (min) sample fresh weight (g) [90].

### 3.4. Determination of Total Cd and Available Cd in Soil and Muskmelon

The content of Cd in plants was extracted by the nitrate method [91]. Leaves of muskmelon at the same node were taken, and root, stem, fruit, and pedicel samples were taken after the fruit matured. Plant tissue and soil samples were dried and ground into fine powder. A total of 3 mL concentrated nitric acid was added to the liquid and boiled in a boiling water bath for 1 h. After cooling, 0.5 mL of 30% H_2_O_2_ solution was added to the reaction system and was heated in a boiling water bath for 0.5 h. The filtrate was cooled and deionized water was added to dilute it to an appropriate concentration. The soil samples were extracted by diethylenetriamine pentaacetic acid (DTPA) and then filtered by shaking table for 2 h and diluted with deionized water. The Cd concentrations in various parts of muskmelon plants and soil were determined by Avanta M atomic absorption spectrophotometer [92].

For heavy metal (HM) pollution, USEPA established a health risk model in 2006. According to the health risk model, a threshold hazard quotient (THQ) was used to evaluate individual non-carcinogenic risk (1). The threshold carcinogenic risk (TCR) is a recognized parameter used to determine an individual’s incremental risk of developing cancer over a lifetime, and the TCR for carcinogenic HMs is calculated using Equation (2) [93].

*THQ* = (*C* × *IngRveg.* × *EF* × *ED*)/(*BW* × *ATc* × *RfD*) (1)

*TCR* = (*C* × *IngRveg.* × *EF* × *ED* × *SF*)/(*BW* × *ATc*) (2)

where *C* is the concentration of plant-heavy metals on the surface (mg/kg), *IngRveg* is the ingestion rate of vegetable (g/day), *EF* is the exposure frequency (day/year), *ED* is the duration of exposure (year), *BW* is body weight (kg), *AT* is the average time (non-carcinogenic risk assessment 365 × *ED*, carcinogenic risk assessment 365 × 70), *RfD* is the reference dose for herbs intake, and *SF* is the cancer slope factor for herbs.

### 3.5. Screening of Extraction Process and Detection of Bioactive Substances

The pedicel was dried and ground into powder, then it was extracted by the ethanol heating extraction method, and the extract solution was obtained after filtration. The optimal extraction range of each extraction factor was determined through single-factor experiments. According to the content of polyphenols, flavonoids, saponins, and polysaccharides in different extraction schemes, three levels were selected for every single factor through the results of single-factor experiments. The content of polyphenols in pedicel was determined by ferrous tartrate spectrophotometry, and the absorbance was detected at 540 nm [94]. The content of flavonoids was determined by aluminum trichloride colorimetry, and the absorbance was measured at 415 nm [95]. The content of saponin was determined by the vanillin-glacial acetic acid method, and the absorbance was measured at 560 nm [96]. The method for determining the content of polysaccharides was the same as described above.

According to the results of the single-factor experiment, each factor selects three levels to design the L9 (_3_^4^) orthogonal experiment table for the orthogonal experiment [97]. Finally, the optimum extraction process was determined. The extract obtained by the optimal extraction process was used to detect the content of active components by UV spectrophotometer, and the effects of different Cd concentrations on the content of bioactive substances were explored.

### 3.6. Cytotoxicity Test

Human cervical cancer cells (HeLa cells) and mouse fibroblast cells (L929 cells) were purchased from China Typical Culture Collection Center (Wuhan, China) [98]. The medium was Dulbecco’s Modified Eagle Medium (DMEM) supplemented with 10% (*v*/*v*) fetal bovine serum and 1% antibody (penicillin and streptomycin) and cultured in a cell incubator at 37 °C and 5% CO_2_.

The muskmelon pedicle extract was lyophilized and then dissolved with a cell culture medium. The cells were treated with different concentrations of drugs, and their activities were determined by CCK8 kit (Biyuntian, Shanghai, China). The HeLa cell suspension was obtained, and 100 μL of the cell suspension (about 4000 cells) was added to a 96-well culture plate and cultured in a cell incubator for 24 h. The medium was discarded and replaced with 100 μL medium containing different concentrations of samples for 24 h. The culture medium was discarded, and 100 μL culture medium and 10 μL CCK8 were added to each well for 2 h. The absorbance at 450 nm was measured by a microplate reader [99]. The L929 cells were treated in the same way as HeLa cells.

Cell inhibition rates were calculated by the following formula: cell proliferation inhibition rate (%) = (OD value of blank control group − OD value of giving medicine group)/OD value of blank control group by 100%.

### 3.7. Statistical Analysis

For each measurement, the value is expressed as mean ± standard deviation (SD). SPSS 22 (SPSS Inc. Chicago, IL, USA) was used for data analysis. Univariate analysis was used for statistical analysis of the experimental data, and Ducan multiple comparisons were used to test the significance of *p* < 0.05. Multiple comparison (LSD-T test) was used for cell inhibition rate data, * means *p* < 0.05, significant; ** means *p* < 0.01, very significant; *** means *p* < 0.001, extremely significant. Software origin 2020 (OriginLab, Northampton, MA, USA) is used for drawing.

## 4. Conclusions

In the study, Cd stress significantly inhibited the growth of melon plants and reduced the contents of chlorophyll and anthocyanin in muskmelon leaves. Cd stress significantly increased the fruit weight, fruit longitudinal diameter, fruit transverse diameter and flesh thickness and increased the sugar content of the fruit. A total of 50 mg/kg Cd induced oxidative stress in muskmelon plants, which increased the activities of anthocyanin and antioxidant enzymes, such as SOD, POD, and APX. When the concentration of Cd increased to 200 mg/kg, the activity of antioxidant enzymes decreased sharply. MDA content increased dramatically, indicating that high Cd stress caused oxidative damage to muskmelon plants. The toxicity of heavy metals to herbal medicine is not only in physiological and biochemical aspects but also in the aspects of bioactive substances. A total of 200 mg/kg Cd increased the content of polyphenol saponins and polysaccharides in the pedicel, while 50 mg/kg Cd inhibited the content of flavonoids. In addition, 50 and 200 mg/kg Cd-stressed extracts of pedicels inhibited the cell activity of normal cells. Under Cd treatment, the cytotoxicity of the crude extract of pedicel to normal cells and cancer cells was lower than that without Cd treatment. The Cd content in all parts is at risk of cancer and exceeds the maximum tolerance of the human body. It is interesting to note in our study that Cd treatment increases the effective components of phenomena, which means that the content of effective components is not representative of the overall quality of bioactive substances in traditional Chinese medicines. It is still necessary to combine the risk assessment of heavy metals, physiological and biochemical tests, and other comprehensive evaluations, which is a problem that relevant departments need to pay attention to. Therefore, the traceability information and quality management of herbs deserve further study.

## Figures and Tables

**Table 1:** Muskmelon fruit size and quality analysis under different treatments. Each treatment was repeated three times, expressed as mean ± standard deviation. Each column with different letters represents significant differences at *p* < 0.05.

Cd Concentration (mg/kg)	Fresh Weight (kg/fruit)	Vertical Diameter (cm)	Transverse Diameter (cm)	Flesh Thickness (cm)	Sugar Content (mg/g)
0	0.204 ± 0.050 b	6.270 ± 0.680 b	7.360 ± 0.608 b	1.017 ± 0.129 b	11.540 ± 2.579 b
50	0.243 ± 0.034 a	6.913 ± 0.512 a	7.890 ± 0.391 a	1.080 ± 0.103 a	12.887 ± 1.424 a
200	0.233 ± 0.035 a	6.820 ± 0.441 a	7.760 ± 0.485 a	1.127 ± 0.120 a	12.483 ± 2.064 ab

**Table 2:** Health risk assessment of different parts treated with different concentrations of Cd. Each treatment was repeated three times, expressed as mean ± standard deviation. Each line of different letters represents significant differences at *p* < 0.05.

Risk	Concentration of Cd	Root	Stem	Leaf	Muskmelon Pedicel	Fruit
THQ	50 mg/kg	658.104 ± 37.332 a	496.859 ± 16.969 b	425.741 ± 0.613 c	106.435 ± 0.661 d	72.087 ± 5.454 e
	200 mg/kg	2505.650 ± 63.892 a	565.144 ± 11.023 b	399.584 ± 6.292 c	113.371 ± 1.811 d	76.654 ± 0.644 d
TCR	50 mg/kg	3.582 ± 0.195 a	2.598 ± 0.089 b	2.223 ± 0.003 c	0.557 ± 0.004 d	0.377 ± 0.029 e
	200 mg/kg	13.101 ± 0.334 a	2.955 ± 0.058 b	2.089 ± 0.033 c	0.593 ± 0.010 d	0.401 ± 0.003 d

## Data Availability

This experiment did not use or analyze any public data, are measured by experiments.

## Supplementary Materials

The following supporting information can be downloaded at: https://www.mdpi.com/article/10.3390/molecules27092913/s1, Table S1: Physical and chemical properties of soil; Figure S1: A, B, and C were 200 mg /kg Cd, 50 mg /kg Cd, and untreated melon (control group); Table S2: Single-factor experiment results of ethanol concentration; Table S3: Extract temperature single-factor experiment results; Table S4: Single-factor experiment results of material–liquid ratio; Table S5: Extraction time of single-factor experiment results; Table S6: Orthogonal test results of extraction of polyphenols, flavonoids, saponins, and polysaccharides from muskmelon pedicel; Table S7: Intuitive analysis of orthogonal test results of the extraction of medicinal components from muskmelon.

## Sample Availability

Muskmelon seeds are available from the authors.
