# Supplementary material for: Effects of Cadmium on Physiochemistry and Bioactive Substances of Muskmelon (Cucumis melo L.)
Source: Molecules. 2022 May 3;27(9):2913. doi: 10.3390/molecules27092913 (PMC9101123; doi:10.3390/molecules27092913)
Supplement: Supplementary file 1 [file molecules-27-02913-s001.zip › molecules-1682071-supplementary.pdf]

*Table S.1 Physical and chemical properties of soil*

| Available Nitrogen (mg/kg) | Available Phosphorus (mg/kg) | Available Potassium (mg/kg) |
|---|---|---|
| 48.84 | 158.92 | 1775.42 |

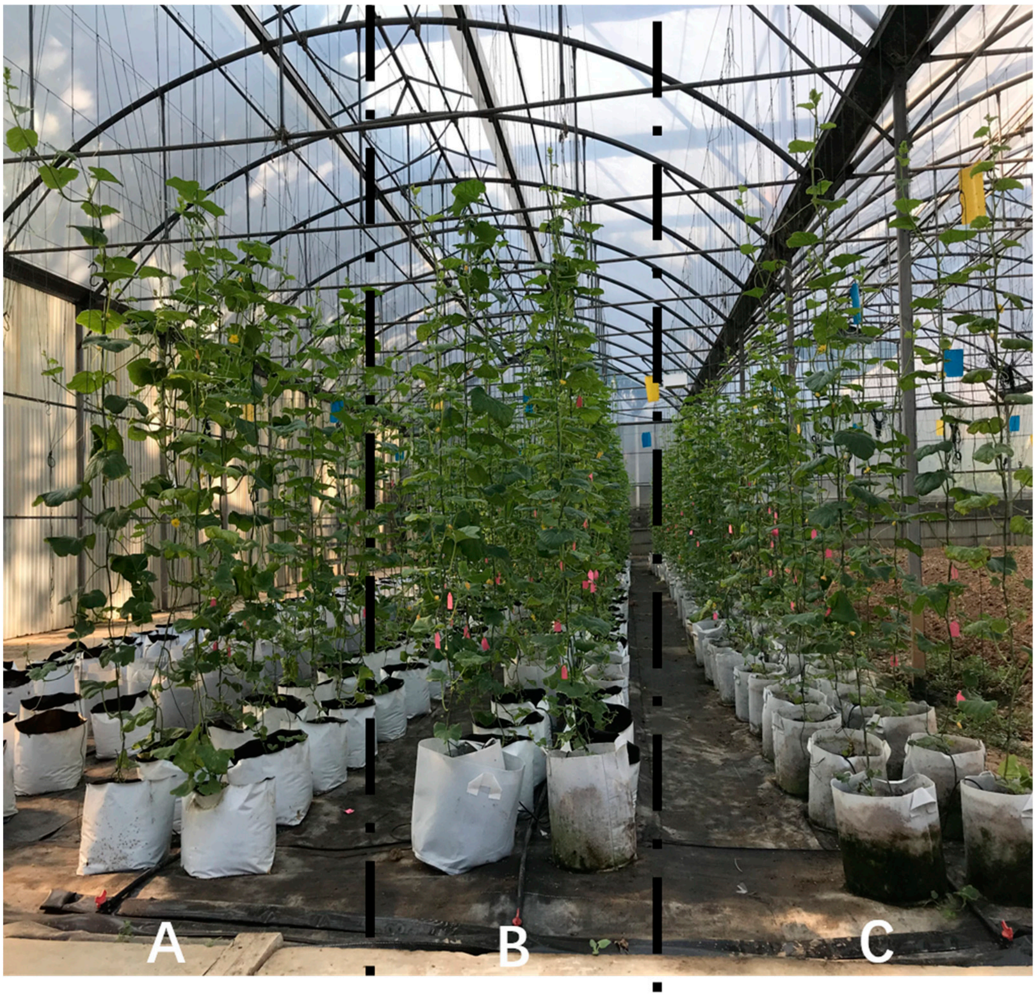


Figure S.1 A, B and C were was 200 mg /kg Cd, 50 mg /kg Cd and untreated melon (control group)

Table S.2 Single factor experiment results of ethanol concentration

| Ethanol concentration | Polyphenols (mg/g) | Flavonoids (mg/g) | Saponin (mg/g) | Polysaccharides (mg/g) |
|---|---|---|---|---|
| 20% | 0.26 E-03 | 0.063 | 0.087 | 181.188 |
| 30% | 0.42 E-03 | 0.052 | 0.060 | 209.297 |
| 40% | 0.75 E-03 | 0.054 | 0.049 | 115.240 |
| 50% | 2.47 E-03 | 0.115 | 0.041 | 97.947 |
| 60% | 2.90 E-03 | 0.116 | 0.036 | 45.272 |
| 70% | 4.17 E-03 | 0.154 | 0.039 | 67.773 |
| 80% | 4.61 E-03 | 0.171 | 0.040 | 39.215 |
| 95% | 5.42 E-03 | 0.204 | 0.068 | 32.295 |

Table S.3 Extract temperature single factor experiment results

| Temperature (℃) | Polyphenols (mg/g) | Flavonoids (mg/g) | Saponin (mg/g) | Polysaccharides (mg/g) |
|---|---|---|---|---|
| 30 | 3.90 E-03 | 0.190 | 0.088 | 40.873 |
| 40 | 3.80 E-03 | 0.198 | 0.085 | 47.109 |
| 50 | 4.32 E-03 | 0.250 | 0.096 | 50.699 |
| 60 | 4.36 E-03 | 0.251 | 0.093 | 43.894 |
| 70 | 4.95 E-03 | 0.276 | 0.118 | 34.534 |
| 80 | 7.55 E-03 | 0.323 | 0.133 | 53.344 |
| 90 | 3.91 E-03 | 0.190 | 0.074 | 42.024 |

Table S.4 Single factor experiment results of material-liquid ratio

| material-liquid ratio | Polyphenols (mg/g) | Flavonoids (mg/g) | Saponin (mg/g) | Polysaccharides (mg/g) |
|---|---|---|---|---|
| 1:10 | 1.50 E-03 | 0.092 | 0.025 | 14.392 |
| 1:20 | 1.58 E-03 | 0.127 | 0.040 | 13.086 |
| 1:30 | 1.80 E-03 | 0.131 | 0.045 | 23.648 |
| 1:40 | 2.72 E-03 | 0.172 | 0.064 | 59.516 |
| 1:50 | 2.79 E-03 | 0.171 | 0.059 | 60.943 |

Table S.5 Extraction time single factor experiment results

| Time (h) | Polyphenols (mg/g) | Flavonoids (mg/g) | Saponin (mg/g) | Polysaccharides (mg/g) |
|---|---|---|---|---|
| 0.5 | 3.99 E-03 | 0.177 | 0.055 | 42.871 |
| 1 | 3.80 E-03 | 0.173 | 0.056 | 30.548 |

| | | | | |
|---|---|---|---|---|
| 2 | 4.40 E-03 | 0.201 | 0.064 | 42.349 |
| 3 | 4.59 E-03 | 0.208 | 0.060 | 48.031 |
| 4 | 4.78 E-03 | 0.213 | 0.063 | 47.346 |
| 5 | 5.35 E-03 | 0.234 | 0.067 | 26.108 |

Table S.6 Orthogonal test results of extraction of polyphenols, flavonoids, saponins and polysaccharides from muskmelon pedicel

| number | A | B | C | D | Polyphenols (mg/g) | Flavonoids (mg/g) | Saponin (mg/g) | Polysaccharides (mg/g) | Comprehensive score |
|---|---|---|---|---|---|---|---|---|---|
| 1 | 1 | 1 | 1 | 1 | 4.73 E-03 | 0.226 | 0.044 | 39.915 | 192.548 |
| 2 | 1 | 2 | 2 | 2 | 6.07 E-03 | 0.249 | 0.044 | 36.670 | 223.619 |
| 3 | 1 | 3 | 3 | 3 | 5.42 E-03 | 0.237 | 0.066 | 35.763 | 221.699 |
| 4 | 2 | 1 | 2 | 3 | 6.72 E-03 | 0.287 | 0.071 | 49.398 | 266.832 |
| 5 | 2 | 2 | 3 | 1 | 4.83 E-03 | 0.201 | 0.089 | 56.686 | 217.350 |
| 6 | 2 | 3 | 1 | 2 | 4.47 E-03 | 0.197 | 0.041 | 44.570 | 177.597 |
| 7 | 3 | 1 | 3 | 2 | 4.37 E-03 | 0.220 | 0.067 | 69.791 | 204.297 |
| 8 | 3 | 2 | 1 | 3 | 5.39 E-03 | 0.243 | 0.084 | 94.279 | 244.581 |
| 9 | 3 | 3 | 2 | 1 | 5.79 E-03 | 0.244 | 0.057 | 85.883 | 233.892 |

Table S.7 Intuitive analysis of orthogonal test results of extraction of medicinal components from muskmelon

| | Polyphenols (mg/g) | | | | Flavonoids (mg/g) | | | |
|---|---|---|---|---|---|---|---|---|
| | A | B | C | D | A | B | C | D |
| k1 | 5.40E-03 | 5.30E-03 | 4.90E-03 | 5.10E-03 | 0.237 | 0.244 | 0.222 | 0.224 |
| k2 | 5.30E-03 | 5.40E-03 | 6.20E-03 | 5.00E-03 | 0.228 | 0.231 | 0.260 | 0.222 |
| k3 | 5.20E-03 | 5.20E-03 | 4.90E-03 | 5.80E-03 | 0.235 | 0.226 | 0.219 | 0.255 |
| R | 2.20E-04 | 2.00E-04 | 1.30E-03 | 8.70E-04 | 0.009 | 0.018 | 0.041 | 0.034 |

Continued

| Saponin (mg/g) | | | | Polysaccharides (mg/g) | | | | Comprehensive score | | | |
|---|---|---|---|---|---|---|---|---|---|---|---|
| A | B | C | D | A | B | C | D | A | B | C | D |
| 0.051 | 0.061 | 0.056 | 0.063 | 37.450 | 53.034 | 59.588 | 60.828 | 212.622 | 221.226 | 204.909 | 214.597 |
| 0.067 | 0.072 | 0.057 | 0.051 | 50.218 | 62.545 | 57.317 | 50.344 | 220.593 | 228.517 | 241.448 | 201.838 |
| 0.070 | 0.055 | 0.074 | 0.074 | 83.317 | 55.406 | 54.080 | 59.813 | 227.590 | 211.063 | 214.449 | 244.371 |
| 0.018 | 0.018 | 0.018 | 0.023 | 45.868 | 9.511 | 5.508 | 10.484 | 14.968 | 17.454 | 36.539 | 42.533 |

K1,k2,k3 represents the average yield at a certain level under a certain factor, respectively. R represents range. A, B, C and D represent ethanol concentration, temperature, liquid-solid ratio and time, respectively
